# Use of a Multiplexed CMOS Microarray to Optimize and Compare Oligonucleotide Binding to DNA Probes Synthesized or Immobilized on Individual Electrodes

**DOI:** 10.3390/s100807371

**Published:** 2010-08-05

**Authors:** Karl Maurer, Nina Yazvenko, Jodi Wilmoth, John Cooper, Wanda Lyon, David Danley

**Affiliations:** 1 CombiMatrix Corporation, 6500 Harbour Heights Parkway, Suite #202, Mukilteo WA 98275, USA; E-Mails: maurer.karl@gmail.com (K.M.); nina.yazvenko@gmail.com (N.Y.); jodildalrymple@yahoo.com (J.W.); jjcooper.seattle@gmail.com (J.C.); ddanley@combimatrix.com (D.D.); 2 AFRL/RHPB, Wright Patterson AFB, 2729 R Street, Area B B837, Dayton, OH 45433, USA; E-Mail: wanda.lyon@wpafb.af.mil

**Keywords:** CombiMatrix microarray, biosensor, electrochemical detection, DNA, hybridization, polypyrrole

## Abstract

The CombiMatrix microarray with 12,544 electrodes supports *in situ* electrochemical synthesis of user-defined DNA probes. As an alternative, we immobilized commercially synthesized DNA probes on individual electrodes coated with electropolymerized polypyrrole (Ppy). Hybridization was measured using a biotinylated target oligonucleotide and either Cy5-streptavidin and fluorescence detection or horseradish peroxidase-streptavidin and enzyme-enhanced electrochemical detection. Detection efficiencies were optimized by varying the deposition of the Ppy, the terminal groups on the DNA probes, and other factors that impacted fluorescence quenching and electrical conductivity. Optimized results were compared against those obtained using a microarray with the same DNA sequences synthesized *in situ*. Immobilized probes produced higher fluorescence signals, possibly by providing a greater stand off between the Cy5 on the target oligonucleotide and the quenching effects of the Ppy and the platinum electrode.

## 1. Introduction

CombiMatrix microarrays were initially developed as highly multiplexed platforms for electrochemistry. The original complementary metal oxide (CMOS) microarray had 1,000 platinum (Pt) electrodes (1K microarray), and it was used to develop the *in situ* electrochemical synthesis of different DNA probes on individual electrodes [1]. Hybridization to these probes was detected using enzyme-enhanced electrochemical detection (ECD) [2]. The second generation microarray with 12,544 electrodes was mounted in a ceramic slide that was designed so that the chip could be read on a commercial fluorescent microarray reader. The 12K CustomArray^®^ microarray is commercially available as a custom gene chip that has been used for a variety of genomic assays (e.g., genotyping, gene expression, SNP analysis, *etc.*). CombiMatrix also developed the ElectraSense^®^ microarray and microarray reader based on ECD. In comparative studies, ECD provides comparable results to fluorescence detection [3,4]. The latest version of the ElectraSense microarray reader is a palm-sized instrument that interfaces with a personal computer through a USB connection, which provides a data link and power to the reader.

The microarray offers unique capabilities for applications where the electrochemical synthesis or deposition of different molecules on electrodes and different methods of detection are required. Tesfu *et al*. [5] and Stuart *el al.* [6] used the 1K microarray to synthesize coumarin or to demonstrate a site-selective hetero-Michael reaction on individual electrodes. Successful execution of these chemistries was determined using fluorescence detection and cyclic voltammetry (CV). Cheng *et al.* [7] reported on using the array with fluorescence detection and time-of-flight secondary ion mass spectrometry to demonstrated molecular synthesis using Wacker oxidations.

We recently reported on using electropolymerization to deposit polypyrrole (Ppy) and adsorb antibodies (Ab) on individual electrodes of the 12 K microarray [8]. This approach was used to develop a very sensitive sandwich immunoassay for staphylococcal enterotoxin B (SEB) using ECD or fluorescence detection. Wojciechowski [9] demonstrated that this array could be used to detect inactivated *Yersinia pestis* and SEB in a multiplex assay.

In this communication, we report on using the microarray with electropolymerized Ppy to immobilize different DNA oligonucleotides on individual electrodes. Immobilizing DNA to electrode surfaces using Ppy was originally reported by Minehan *et al.* [10]. Since that finding, numerous studies have been done using this and other electroactive polymers as described in recent reviews [11–17]. Most of the studies reported on using label less detection (e.g., CV and electrochemical impedance spectroscopy) for measuring DNA hybridization. More relevant to our findings are those reported by investigators at CIS Bio international and CEA [18–22]. This group developed a CMOS microarray with 128 addressable electrodes, and they co-polymerized pyrrole with pyrrole-conjugated DNA probes to create a multiplexed gene chip for the fluorescence detection of hybridization. Unique to this communication, we have measured hybridization using ECD and fluorescence detection on the same platform. Detection efficiencies were optimized by varying the deposition of the Ppy, the terminal groups on the DNA probes, and other factors that impacted on fluorescence quenching and electrical conductivity. Optimized results were compared against those obtained using a microarray with the same DNA sequences synthesized *in situ*. Immobilized probes produced higher fluorescence signals, possibly by providing a greater stand off between the Cy5 on the target oligonucleotide and the quenching effects of the Ppy and the platinum electrode.

## 2. Experimental Section

### 2.1. Reagents

Biotinylated oligonucleotide and DNA probes were purchased from Integrated DNA Technologies (Coralville, IA). The sequence of the labeled DNA target is 5′-biotin TGC-TTC-TGT-ACG-TTG-TAC-CCA, the sequence for the complementary DNA probe is 5′-TGG-GTA-CAA-CGT-ACA-GAA-GCA, the sequence of the non complementary DNA probe is 5′-CAA-TAG-CTC-CTG-CTA-CAA-ATG-C. Probes were labeled at their 5′-ends with an amine, a disulfide, or a 20 T-linker with an amine. Prior to immobilization on the Ppy, the disulfide DNA was diluted in phosphate buffered saline (PBS) to 0.40 mg/mL and mixed with an equal volume of Immobilized TCEP Disulfide Reducing Gel in PBS (Thermo Fisher Scientific, Rockford, IL). The mixture was shaken at 25 °C for 1 h. Following low speed centrifugation, the supernatant was recovered; and the gel was washed once with PBS, which was pooled with the original supernatant to yield a final DNA concentration of 0.20 mg/mL. The thiol-terminated DNA was used immediately to prevent reformation of disulfide bonds. The protein blocking solution (PBSC) and pyrrole were prepared as described previously [8]. Propanolamine, cysteine, and thioglycolic acid (Sigma-Aldrich, St. Louis, MO) blocking solutions were prepared by suspending each in PBS (pH 7.4) to a concentration of 1.0 M.

### 2.2. Methods

*Immobilization of DNA Probes on Individual Electrodes.* Two methods were used for immobilizing DNA probes on individual electrodes. The first method involved *in situ* synthesis using the CombiMatrix commercial process [1]. The second method involved deposition of Ppy and DNA probes using the same procedure described previously for Ab immobilization [8]. In short, a chip map was created for the PotentioSense and MX300 instruments by designating through the software which electrodes were to be addressed, the current to be applied, and the time of application. The map created four replicated areas on the array that corresponded to the four chambers of a plastic hyb cap (ElectraSense Hybridization Cap, 4 × 2 K, CombiMatrix Corp., Mukilteo, WA). Within each area, 2 × 2 blocks of electrodes were connected through CMOS transistor switches on the array so that they received the same current for the same period of time. To prevent non-specific binding, the array was treated with PBSC for 5 min, washed three times with PBS containing 0.1% Tween 20 (PBST), three times with PBS, and three times with 0.1 M dibasic sodium sulfate prior to adding pyrrole for electrodeposition. After Ppy deposition, the array was washed twice with PBS; and the DNA oligonucleotide, diluted in PBS, was added for 15 min at 25 °C. The array was washed three times with PBSC and blocked with the same for 2–5 min. For deposition of a second oligonucleotide, the array was washed thrice with PBST, with PBS and with sodium sulfate prior to Ppy deposition as described above. After probe deposition, the microarray was blocked with PBSC for 1 h, and stored at 4 °C. To inhibit thiol-DNA immobilization, Ppy was deposited as described, and the array was washed twice with PBS and incubated for 15 min at 25 °C in the dark with a blocking solution. The array was washed three times with PBS, and the thiol-DNA was deposited in the prescribed manner.

*Microarray Hybridization.* Hybridizations were done manually so that results from experiments using ECD and fluorescence detection were processed in the same manner. The microarray was fitted with a four-chamber hyb cap and washed with PBSC before adding a dilution of biotinylated DNA target in 2XPBST or 2XPBST alone (control). Following a 1 h incubation at 50 °C, the chambers were washed three times with 2XPBST, the four-chambered hyb cap was removed and replaced with a single-chambered hyb cap, and the array was washed three more times. The array was incubated with 5XPBSC (BioFX, Owings Mills, MD) for 20 min at 25 °C and washed three times with 2XPBST. For fluorescence detection, microarrays were incubated for 30 min with Cy5-streptavidin (GE Healthcare, Amersham Biosciences, Piscataway, NJ diluted to 1.0 μg/mL in 2XPBST. Arrays were washed five times in PBSC, twice in PBS, and scanned on a GenePix 4000B (Axon Instruments, Molecular Devices, Sunnyvale, CA). For ECD, microarrays were incubated for 30 min with Poly-80-HRP Streptavidin (Fitzgerald Industries International, Acton, MA) diluted 1:1,000 in PBST. Arrays were washed four times with PBSC, once with PBS, and twice with pH 4 Conductivity Buffer Substrate (BioFX). TMB Conductivity 1 Component HRP Microwell Substrate (BioFX) was added to the array, and it was scanned immediately with an ElectraSense microarray reader (CombiMatrix Corp.). Data were quantified using Microarray Imager or ElectraSense software (CombiMatrix Corp.) for fluorescent scans or ECD respectively.

## 3. Results and Discussion

**Figure 1.**
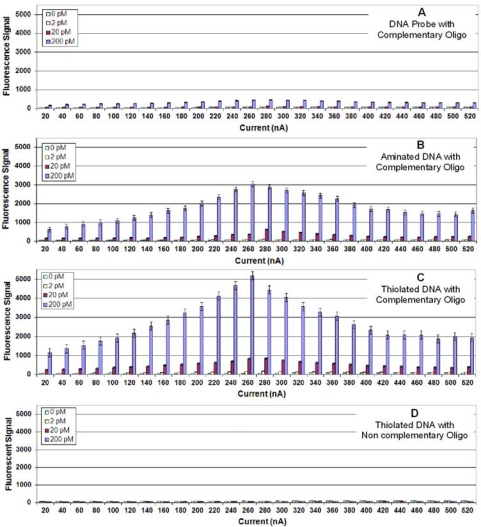
**(A)** Fluorescence detection of target oligonucleotide binding to a complementary probe immobilized on Ppy deposited using constant current from 10 to 520 nA for 1.0 s. Different concentrations (0, 2, 20 or 200 pM) of target oligonucleotide were incubated in individual chambers of a four-chamber hyb cap, and binding was detected using Cy5-SA. **(B)** Same as (1A), but a 5′-aminated complementary probe was immobilized on the Ppy. (**C**) Same as (1A) but a 5′-thiolated complementary probe was immobilized on the Ppy. (**D**) Same as (1A) but a 5′-thiolated non-complementary probe was immobilized on the Ppy.

In our earlier study on fixing Ab to Ppy and detecting antigen binding, we observed that Ppy deposition conditions (current and time) influenced assay results; and the conditions that favored optimum ECD were different than those that favored optimum fluorescence detection [8]. For studying DNA immobilization on Ppy, we used the same assay protocols and studied the same variables with changes made to optimize detection of DNA hybridization. Figure 1A illustrates the results from fluorescence detection of DNA hybridization to a complementary, unmodified DNA probe (*i.e.*, no 5′ terminal modification) fixed onto the surface of the Ppy. Considering the maximum amount of target oligonucleotide (200 pM) used in the assay, the hybridization signals were low with the optimum signals on Ppy deposited at 260 nA for 1 s.

A number of investigators have relied on entrapment to immobilize unmodified DNA to Ppy; however, more have modified the DNA, the Ppy, or both to create a covalent attachment between one end of the DNA (usually the 5′-end) and the Ppy. This provides a secure and oriented fixation of the DNA to the Ppy that is often illustrated as a lawn of vertical strands standing perpendicular to the Ppy [14]. Figure 1B illustrates the results from target hybridization to a complementary probe with a 5′-terminal amine. Compared with the unmodified DNA, the aminated DNA probe produced almost eight times the signal. A greater than ten-fold increase was obtained when a complementary thiol-DNA probe was used (Figure 1C). The negative control using a thiolated non complementary probe (Figure 1D) produced a negligible background hybridization signal.

**Figure 2.**
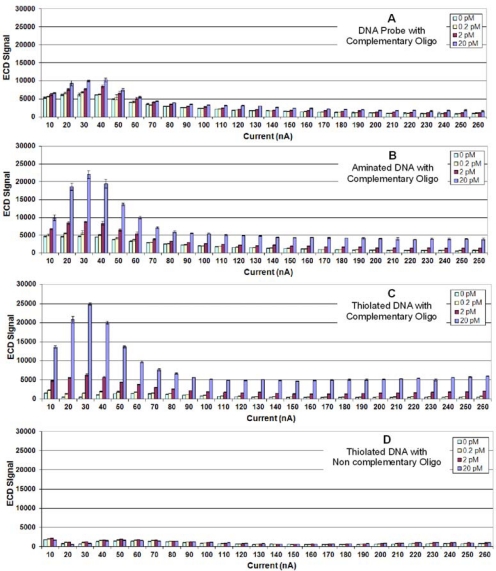
**(A)** Electrochemical detection of target oligonucleotide binding to a complementary DNA probe immobilized on Ppy deposited using constant current from 10 to 260 nA for 1.0 s. Different concentrations (0.0, 0.2, 2.0 or 20.0 pM) of target oligonucleotide were incubated in individual chambers of a four-chamber hyb cap, and binding was detected using HRP-SA. **(B)** Same as (2A), but a 5′-aminated complementary probe was immobilized onto the Ppy. (**C**) Same as (2A) but a 5′-thiolated complementary probe was immobilized on the Ppy. (**D**) Same as (2A) but a 5′-thiolated non-complementary probe was immobilized on the Ppy.

This experiment was repeated using ECD, and Figures 2A–D illustrate the results. As observed using fluorescence detection, aminated and thiolated probes produced much higher hybridization signals (2–2.5 times) than unmodified DNA. However, for ECD, maximum hybridization signals were observed using Ppy deposited at 30 nA; and very high ECD signals were obtained using one tenth the concentration of labeled target.

**Figure 3.**
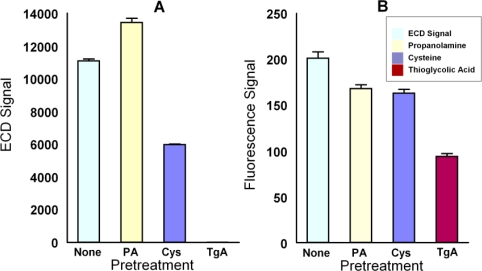
Inhibition of hybridization signals by Ppy pretreatment with 1.0 M propanolamine, cysteine, or thioglycolic acid prior to immobilization of thiolated DNA. (**A**) Effect on ECD measured on electrodes with Ppy polymerized at 40 nA following hybridization with 20 pM 5′-biotinylated complementary oligonucleotide. (**B**) Effect on fluorescence detection, measured on electrodes with Ppy polymerized at 260 nA and hybridized with 200 pM of complementary oligonucleotides.

These results raised two issues—the importance of terminal groups on DNA for binding to Ppy and the relationship between conductivity and fluorescence quenching. With respect to the first, Minehan *et al.* [23] and Gambhir *et al.* [24] reported that the binding of DNA to Ppy is consistent with electrostatic adsorption between the fixed negatively charged phosphates forming the backbone of the DNA and the mobile positively charged defect structures of the Ppy, which favor hydrogen bonding between the phosphates and Ppy ring nitrogen atoms. However, De Giglio *et al.* [25] demonstrated that cysteine binds to Ppy electropolymerized on platinum or titanium electrodes. They presented evidence from X-ray photoelectron spectroscopy that cysteine forms a covalent bond through its sulfur atom by nucleophilic attack on the positive sites of the pyrrole ring. More recently, Zhou *et al.* [26] reported on immobilizing 5′cys-terminated DNA probes to electropolymerized polyaniline via a nucleophilic substitution reaction and measuring hybridization using CV. To determine whether or not the binding of the thiolated DNA probes is mediated through the mechanism described by De Giglio *et al.*, microarrays with electropolymerized Ppy were incubated for 15 min at room temperature with either PBS, or 1.0 M propanolamine, 1.0 M cysteine, or 1.0 M thioglycolic acid in PBS, after which 5′-thiolated complementary DNA was deposited as usual. Hybridization was measured using 200 pM or 20 pM DNA target and fluorescence detection or ECD respectively. Figure 3 shows that cysteine and thioglycolic acid reduced both fluorescence and ECD signals with the latter demonstrating excellent effectiveness in both assays. Pretreatment of the Ppy with propanolamine had mixed effects on the assay by increasing the signal as measured by ECD while decreasing the signal as measured by fluorescence. This suggests that propanolamine affected some quality of the Ppy (e.g., conductivity) that may not be related to blocking oligonucleotide binding.

With respect to the apparent inverse relationship between Ppy conductivity and fluorescence quenching, we did not observe the latter in developing an immunoassay on the array [8]. However, Ramanvicius *et al.* [27] used Ppy fluorescence quenching to develop an immunoassay against bovine leukemia virus protein gp51. They attributed the quenching to the proximity of the Cy5 to the delocalized π-π electrons in the Ppy backbone, as described by Song *et al.* [28]. Livache *et al.* [19] did not describe fluorescence quenching by Ppy in their development of a DNA chip that used phycoerythrin as the fluorescent marker; however, they did note that fluorescence increased with increasing Ppy thickness and with a T-linker of increasing length between the pyrrole and the oligonucleotide 5′ end. The Ppy thickness used by these investigators was 20 nm, which was produced by dipping the electrode in 20 mM pyrrole with 1 μM pyrrole-conjugate oligonucleotide and electro-copolymerizing them using CV until a charge of 250 nC was reached. This charge value is close to the optimum range we observed using constant current for Ppy electropolymerization on our 43 μ Pt electrodes (260 nA for 1 s).

**Figure 4.**
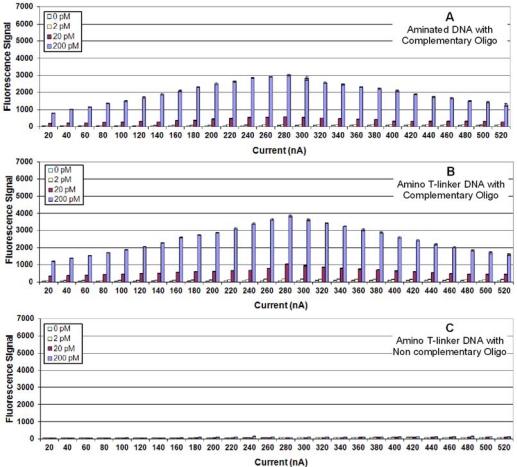
**(A)** Fluorescence detection of target oligonucleotide binding to a complementary aminated DNA probe immobilized on Ppy deposited using constant current from 10 to 520 nA for 1.0 s. Different concentrations (0, 2, 20, or 200 pM) of 5′-biotinylated target oligonucleotide were incubated in individual chambers of a four-chamber hyb cap, and binding was detected using Cy5-SA. **(B)** Same as (3A), but a complementary DNA probe with a 5′-aminated T-linker was immobilized on the Ppy. (**C**) Same as (3A), but a non-complementary DNA probe with an aminated T-linker was immobilized on the Ppy.

To determine if extending the probe further from the surface of the Ppy would change the fluorescence signal, we added a 20 T-linker between the 5′-end and the terminal amine (aminated T-linker). Figure 4 illustrates that the probe with the aminated T-linker showed a 33% increase in fluorescence hybridization signals compared to signals obtained using the aminated DNA probe without the linker.

**Figure 5.**
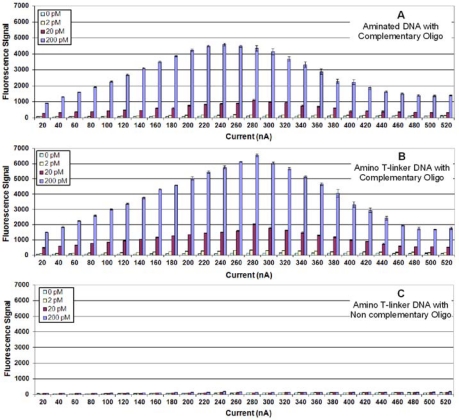
Fluorescence detection of rehybridization by target oligonucleotide to probes on the microarray used in Figure 3 following stripping at 95 °C for 1 h. Different concentrations (0, 2, 20, or 200 pM) of biotinylated target oligonucleotide were incubated in individual chambers of a four-chamber hyb cap, and binding was detected using Cy5-SA. (**A**) Complementary 5′-aminated DNA probe immobilized on Ppy deposited using constant current from 10 to 520 nA for 1.0 s. **(B)** Same as (4A), but a complementary DNA probe with an 5′-aminated T-linker was immobilized on the Ppy. (**C**) Same as (4A), but a non-complementary DNA probe with a 5′-aminated T-linker was immobilized on the Ppy.

In the course of these studies, we stripped the microarrays for reuse by incubating them in PBS at 95 °C for 1 h. Figure 5A–C illustrates the fluorescence signals obtained after stripping the microarray that was used for studies reported in Figure 4 and rehybridizing it with 5′-biotinylated oligonucleotide. Stripping, removed all fluorescence, and it could not be reconstituted by labeling with Cy5-SA alone (data not shown). However, upon rehybridization and labeling, the fluorescence signals were 50 to 70% higher than in the original hybridization. To ensure that this enhancement was not related to hybridization and stripping, we heated a microarray with immobilized probes to 95 °C for 1 h prior to hybridization and obtained comparable results (data not shown). Moreover, stripping or preheating the microarrays had negligible effect on hybridization to the non-complementary DNA probes (Figure 5C).

**Figure 6.**
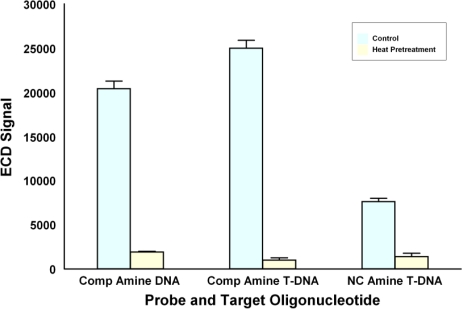
Effects on ECD of adding a 5′-aminated 20 T-linker to DNA probes and preheating the immobilized probes prior to hybridization. Polypyrrole was deposited at 30 nA, and 20 pM of biotinylated oligonucleotide was hybridized on the array. A second microarray was incubated in 2XPBST for 1 h at 95 °C and washed once in PBS prior to hybridization.

These studies were repeated using ECD and Figure 6 shows that adding an aminated T linker to the DNA probe increased hybridization signals by 22%; however, heating the microarray prior to hybridization reduced the ECD signal to background levels. The opposite effects of heating on fluorescence detection and ECD suggest that heating may be changing the nature of the Ppy as opposed to altering the DNA probes. Neoh *et al.* [29] and Ando *et al.* [30] reported that elevated temperatures (100–200 °C) reduced the conductivity of Ppy through a number of possible mechanisms. Reduced conductivity would reduce ECD signals while improving fluorescence signals by reducing quenching—a function of conductivity [28].

**Figure 7.**
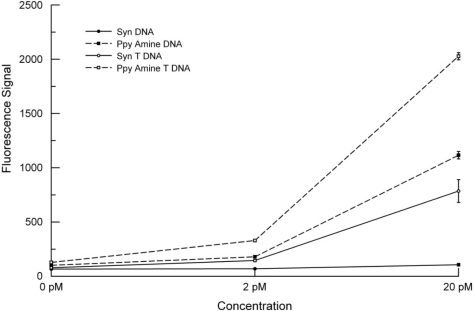
Concentration of target *versus* signal intensity plot for two microarrays containing complementary and non complementary DNA probes either synthesized (Syn) *in situ* or immobilized on polypyrrole (Ppy). The data illustrate results using a synthesized complementary DNA probe (Syn DNA), a synthesized complementary DNA probe with a 3′ 20 T-linker (Syn T DNA), a complementary 5′ aminated DNA probe on Ppy (Ppy Amine DNA), and a complementary DNA probe with a 5′ aminated T-linker (Ppy Amine T DNA). Microarrays were hybridized with 0, 2, or 20 pM of biotinylated oligonucleotide.

**Figure 8.**
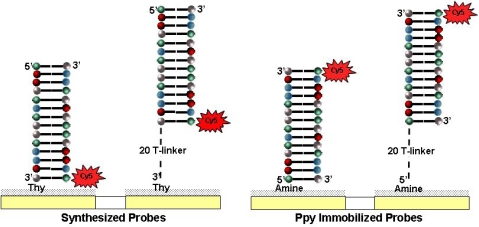
Illustration of the relationship between the Cy5 dye on the target oligonucleotide and the Pt or Ppy surface on the electrode for the DNA capture probes either synthesized *in situ* or immobilized using Ppy respectively.

Because oligonucleotides can be synthesized on the microarray, we produced an array that contained probes with and without 20-T linkers and in the same configuration as the Ppy arrays. Figure 7 compares results from a synthesized microarray against one prepared using Ppy that was pretreated with heat to obtain maximum hybridization signals. The highest hybridization signals were obtained using the complementary probe with aminated T-linker on Ppy, followed by the aminated DNA probe on Ppy and the synthesized DNA probe with a 20T-linker. The lowest hybridization signals were obtained with the synthesized DNA probe. While these differences may be due to a number of factors, the data suggest an interesting correlation between the intensities of the fluorescence signals and distances between the Cy5 and the quenching surface (Ppy or Pt). As illustrated in Figure 8, *in situ* DNA synthesis occurs 3′ to 5′, which means that an oligonucleotide labeled on its 5′-end will hybridize with the Cy5 next to the Pt electrode. Adding a 20 T-linker will move the Cy5 away from the membrane by 20 bases. The aminated DNA is tethered to the Ppy by its 5′-end, and the target oligonucleotide hybridizes with the Cy5 in the opposite orientation and 21 bases away from the Ppy—about the same distance as synthesized DNA with a 20 T-linker. The aminated T-linker DNA adds another 20 bases on the 5′-end, which puts the Cy5 the furthest away (41 bases) from the Ppy. However, this model is predicated on a uniform lawn of DNA standing perpendicular to the surface. Other factors may also have a bearing on these results, e.g., differences in the surface densities of the DNA probes, steric hindrance of hybridization [8] and/or labeling, and possibly DNA electroconductivity [31].

## 4. Conclusions

The results presented herein substantiate and contribute to the observations reported in a variety of publications regarding the use of Ppy as an electroactive membrane for the deposition of biological molecules on electrodes. Unique to this report, however, is the use of fluorescence detection and ECD on the same experimental platform and an empirical approach for identifying factors that influence the performance of each. Fluorescence detection relies on the measurement of emitted photons resulting from the stimulation of a fluorescent molecule by a high energy light source (e.g., laser). Detection of the emitted light at each electrode (feature) on the array requires an instrument with a stable optical system, detector, and software to create a microarray image. Enzyme-enhanced electrochemical detection uses a redox molecule and substrates to produce electrons that are measured through the electrode, the CMOS circuitry of the array and computer software. Compared with a fluorescent microarray scanner, ECD detectors are much simpler, smaller, more robust, and less expensive. However, fluorescent scanners are widely used because they can accommodate different microarray platforms.

Others have used the 12K microarray to compare ECD with fluorescence detection of hybridization using *in situ* synthesized DNA probes [3,4], and they have determined that both methods of detection work equally well However, when DNA probes are adsorbed onto electropolymerized Ppy on this array, significant differences are apparent. For ECD, optimum hybridization signals were obtained when a thin layer of Ppy was applied (30 nA for 1 s), whereas for fluorescence detection a thicker layer gave higher hybridization signals (260 nA for 1 s). These optimum conditions for Ppy deposition and DNA hybridization detection are the same as we observed previously for detecting Ab/Ag binding using ECD and fluorescence detection respectively [8]. While Ab deposition did not require chemical modification to the capture molecule, terminating the DNA probe with an amine or thiol group improved both methods of detection, possibly by promoting the formation of covalent bonds between the DNA probe and nucleophilic centers in the Ppy. Nevertheless, ECD was ten times more sensitive than fluorescence detection, which appears to be the result of fluorescence quenching by the Ppy. Fluorescent signals were improved by extending the capture probe using a T-linker and by heating the array to 95 °C for 1 h prior to hybridization. Heating improved the fluorescence signal and reduced the ECD signal, indicating that it was affecting the Ppy rather than the immobilized DNA probes, possibly by reducing the conductivity of the former. Pretreatment of Ppy with propanolamine had the opposite effect—the ECD signal improved while the fluorescence signal decreased. Comparing the hybridization signals using probes that were synthesized situ *versus* those immobilized on Ppy, we observed higher fluorescence signals from the latter. While differences appear to be related to the proximity of the fluorescent dye to the quenching effect of the Pt electrode or the Ppy, there are other factors that could influence these results as well. The versatility of the 12K microarray to support different methods for depositing capture elements (DNA and Ab) and different methods for detecting target binding creates opportunities for developing multiplex assays that use orthogonal methods to identifying desired target molecules including but not limited to protein, peptides, organisms, and nucleic acid biomarkers.
